# Enhancing immunotherapy efficacy in multiple myeloma and chronic lymphocytic leukemia: from combinatorial therapeutic approaches to gut microbiota modulation

**DOI:** 10.3389/fimmu.2026.1805129

**Published:** 2026-06-17

**Authors:** Andrea Adrados, Juan Pérez-Fernández, Roberto García-Vicente, Alba Garrote-de-Barros, Joaquín Martinez-Lopez, María Linares, María Hernández-Sánchez

**Affiliations:** 1 Department of Translational Hematology, Instituto de Investigación Hospital 12 de Octubre (imas12), Hematological Malignancies Clinical Research Unit H12OCNIO, Madrid, Spain; 2 Department of Biochemistry and Molecular Biology, Pharmacy School, Universidad Complutense de Madrid, Madrid, Spain; 3 Department of Medicine, Faculty of Medicine, Complutense University of Madrid, Madrid, Spain

**Keywords:** chronic lymphocytic leukemia, combinatorial therapies, gut microbiota, immunotherapy, multiple myeloma

## Abstract

Multiple myeloma and chronic lymphocytic leukemia are among the most common hematological tumors. Although first-line therapies are initially effective, their clinical benefit is frequently compromised by the emergence of acquired drug resistance. In recent years, novel immunotherapies such as checkpoint inhibitors, chimeric antigen receptor T-cells, and bispecific antibodies are showing promising results in preclinical studies, achieving high rates of response and remission in some cases. However, despite these advances, durable responses are not achieved in all patients, and treatment failure, toxicity and resistance remain major clinical challenges. In this review, we have comprehensively examined current strategies aimed at optimizing immunotherapy efficacy through rational combination approaches including novel immunotherapies with established standard-of-care agents as well as emerging treatments. Moreover, we have explored the growing evidence supporting the role of the gut microbiota in both B-cell malignancies, focusing on its influence on disease biology and modulation of immunotherapeutic responses to further improve patient outcomes. Finally, we have addressed the current challenges and future perspectives in the field of immunotherapy in both diseases.

## 1 Introduction

### 1.1 B-cell malignancies

Malignant B-cell neoplasms constitute a heterogeneous group of hematological tumors that arise from various alterations during different stages of B-cell differentiation (1), leading to the uncontrolled proliferation of single B cells at different stages of maturation. These neoplasms retain phenotypic and functional characteristics of their cell of origin, particularly in terms of differentiation status and proliferative capacity (2). B-cell malignancies predominantly involve the peripheral blood, bone marrow, and lymphoid tissues, and encompass B-cell lymphomas, B-cell leukemias, and plasma cell dyscrasias (3). The classification of these neoplasms has been refined by the World Health Organization’s 5th edition of the classification of hematolymphoid tumors (WHO-HAEM5, 2022) (4).

#### 1.1.1 Multiple myeloma

Plasma cell neoplasms represent a group of malignancies originating from terminally differentiated B cells and are typically characterized by the secretion of a class-switched monoclonal immunoglobulin (5). Multiple myeloma (MM), or plasma cell myeloma, is defined by the clonal proliferation and accumulation of malignant plasma cells within the bone marrow, although in advanced stages, these cells may infiltrate peripheral blood, soft tissues, and other organs (6, 7). In most patients, malignant plasma cells produce a monoclonal immunoglobulin (M-protein) (8, 9). Nearly all MM cases arise from an asymptomatic precursor state, monoclonal gammopathy of undetermined significance (MGUS), which affects approximately 3% of individuals over 50 years old (10, 11) and progresses to MM at roughly 1% per year (11). An intermediate, asymptomatic stage known as smoldering multiple myeloma (SMM) may also occur, with an annual progression rate to MM of ~10% during the first five years (12, 13).

MM has an incidence of 2.8–4.8 per 100, 000 individuals, accounting for 1.7% of all cancers and 10% of hematologic malignancies in developed countries (6, 14). It primarily affects older adults, with a median age at diagnosis of 69 years, and shows a higher prevalence in men and in African American and Black populations (15, 16). The 5-year overall survival rate is approximately 61% (7). MM remains incurable but treatable, with therapy aimed at prolonging survival and achieving complete responses (17). Treatment choice for newly diagnosed multiple myeloma (NDMM) patients is guided by autologous stem cell transplant (ASCT) eligibility and risk stratification (18). Induction therapy for transplant-eligible (TE) patients typically involves triplet or quadruplet regimens combining proteasome inhibitors, immunomodulatory drugs (IMiDs), corticosteroids, and anti-CD38 antibodies, followed by ASCT, consolidation, and maintenance therapy (19–23). Non-transplant-eligible patients receive tailored combinations of similar agents (24–26). For relapsed or refractory MM (RRMM), treatment is individualized based on prior therapy and drug sensitivity, employing varied combinations of IMiDs, proteasome inhibitors, monoclonal antibodies, CAR-T cell therapies, or bispecific antibodies (27–30).

#### 1.1.2 Chronic lymphocytic leukemia

Chronic lymphocytic leukemia (CLL) is defined as a lymphoproliferative disorder, as a result from the overgrowth of a single CD5+ B lymphocyte co-expressing low levels of surface membrane immunoglobulin (smIg) of a single IG light (L) chain type and of CD79b, CD20, and CD23; involving peripheral blood (PB), bone marrow (BM) and lymphoid organs (31, 32). CLL is the most common leukemia in developed countries and over successive decades advances in therapy have resulted in improvements in survival (33). The diagnosis of CLL requires the presence of ≥5 x10^9^/L clonal B cells of typical phenotype in the blood. Patients with <5 x10^9^/L circulating CLL-type cells may be diagnosed with small lymphocytic lymphoma (SLL) if they also present with either lymphadenopathy, organomegaly or extramedullary disease; or with monoclonal B-cell lymphocytosis (MBL) if they do not (34). The disease may have a stable course but also become aggressive, with frequent relapses, or even transform into an aggressive lymphoma called Richter transformation (RT) (35, 36). Over the past ten years, CLL treatment has seen significant development. The combination of traditional chemotherapeutic drugs with a monoclonal antibody like rituximab or obinutuzumab, was once the basic form of treatment. However, this is no longer the case, at least for most patients. The main therapies currently approved by both US Food and Drug Administration (FDA) and the European Medicines Agency (EMA) for the treatment of CLL include targeted agents such as Bruton’s tyrosine kinase inhibitors (BTKi) and the BCL-2 inhibitor venetoclax as first and second-line therapies. Covalent BTKi ibrutinib and the second-generation inhibitors acalabrutinib and zanubrutinib, are generally administered as continuous regimens until disease progression or unacceptable toxicity. For relapsed/refractory CLL (rrCLL), the non-covalent BTKi pirtobrutinib is also approved. Venetoclax represents another mainstay therapy and is administered in fixed-duration regimens in combination with the anti-CD20 monoclonal antibody obinutuzumab as first-line therapy or rituximab in relapsed/refractory disease. PI3K inhibitors such as duvelisib and idelalisib have been approved by both EMA and FDA, however, their side effects have strongly reduced their usage and stopped the further development of this class of agents for the treatment of CLL (37). While not currently part of standard treatment for most patients, emerging immunotherapeutic approaches in CLL remain largely investigational and are mainly being evaluated in clinical trials. Nevertheless, CAR-T cell therapy currently represents the main regulatory exception, having received FDA approval for selected patients with relapsed/refractory CLL/SLL.

### 1.2 Immunotherapy in B-cell malignancies

#### 1.2.1 Immune checkpoint inhibitor therapy

Immune checkpoints are inhibitory immunoreceptors that act as essential regulators of T-cell activity, functioning as “gatekeepers” that prevent excessive or uncontrolled immune responses (38). Under physiological conditions, T-cell activation requires two signals: antigen recognition through the T-cell receptor (TCR) and a co-stimulatory signal. In the absence of co-stimulation, T cells enter a state of anergy in which they fail to respond to stimulation. Once the immune response has been initiated, inhibitory pathways are activated to restore homeostasis and prevent tissue damage (39).

Among the best-characterized immune checkpoints are programmed cell death protein 1 (PD-1) and cytotoxic T-lymphocyte–associated protein 4 (CTLA-4). PD-1 engagement by PD-L1 downregulates T-cell metabolism and effector function, whereas CTLA-4 competes with CD28 for B7 ligands, limiting T-cell activation and suppressing ongoing immune responses. In recent years, additional inhibitory receptors—such as LAG-3, TIM-3, TIGIT, and BTLA—have been identified as further contributors to T-cell suppression within the tumor microenvironment (39). Therapeutically, antibodies that block these ligand–receptor interactions can release these inhibitory brakes, thereby enhancing antitumor immune responses (40). Immune-checkpoint inhibitors (ICIs) were first approved in 2011 for the treatment of unresectable advanced melanoma following failure of conventional therapies. Over the past decade, these agents have transformed the management of advanced solid tumors, progressively shifting from second- or third-line options to standard first-line treatments (38). Importantly, in hematological malignancies, PD-1 inhibitors including nivolumab and pembrolizumab have been approved for the treatment of relapsed/refractory Hodgkin lymphoma, showing meaningful activity in this setting (39).

Their use is associated with a distinct spectrum of toxicities linked to their mechanism of action, which differs substantially from that of traditional systemic therapies. These immune-related adverse events (irAEs) can affect virtually any organ system, although gastrointestinal, dermatologic, hepatic, endocrine, and pulmonary toxicities are the most frequently observed. The incidence, severity, and timing of irAEs vary depending on the class and dose of the immune checkpoint inhibitor, the underlying tumor type, and patient-specific factors (41).

In MM, several immune checkpoint blockade strategies have been evaluated across clinical trials. These include antibodies targeting CTLA-4 (tremelimumab) (42), PD-1 (pembrolizumab, nivolumab, cemiplimab) (43–45) and PD-L1 (atezolizumab, durvalumab) (46, 47). Furthermore, the therapeutic potential of inhibiting additional immunoregulatory pathways—such as CD47, LAG-3, TIGIT, or killer immunoglobulin-like receptors (KIRs)—has also been explored (48). However, no ICI has received regulatory approval for use in MM. Furthermore, FDA holds clinical trials investigating ICIs in this disease led to reduced patient enrolment and limited the available data on their potential efficacy (49).

CLL alters systemic immunity and can induce T-cell exhaustion, which likely limits the efficacy of ICIs in patients with CLL (50, 51). Consistently, clinical studies evaluating monotherapy PD-1/PD-L1 blockade in CLL have shown minimal therapeutic benefit to date (52, 53). By contrast, ICIs have opened new horizons of treatments in RT patients since these patients present limited responses to targeted therapies (52, 54). For example, pembrolizumab has a selective activity in CLL patients with RT showing more durable responses than in *de novo* diffuse large B-cell lymphoma (DLBCL) (55, 55). At present, most of clinical trials with ICIs are evaluating their efficacy in combination with other CLL-approved drugs such as BTK or BCL-2 inhibitors.

#### 1.2.2 CAR-T cells

Chimeric antigen receptors (CARs) are synthetic receptors composed of three main domains (56, 57). The extracellular domain typically consists of a single-chain variable fragment (scFv) derived from monoclonal antibodies, connected via a hinge to the transmembrane domain, which stabilizes the receptor and anchors it to the T-cell membrane (58, 59). The intracellular domain contains a T-cell activation motif (CD3ζ) and a co-stimulatory domain (CD28 or 4-1BB, among others), enabling downstream signaling upon antigen engagement (58). CAR-T cells are generated by modifying a patient’s own T cells in an autologous context to minimize graft-versus-host disease (58). Peripheral blood T cells are collected via leukapheresis (60), enriched, and activated (61). CAR constructs are introduced using viral vectors (lentiviral or retroviral), followed by *in vitro* expansion under GMP conditions to reach therapeutic doses (62). Prior to infusion, patients undergo lymphodepletion (typically fludarabine and cyclophosphamide) to enhance CAR-T cell expansion and persistence (58, 63, 64). Upon infusion, CAR-T cells recognize tumor antigens via the scFv, triggering receptor clustering and activation through CD3ζ and co-stimulatory signaling persistence (58, 65, 66).

Activated CAR-T cells mediate cytotoxicity via perforin/granzyme release, Fas ligand-induced apoptosis, and secretion of pro-inflammatory cytokines that amplify the antitumor response (66, 67). CAR-T therapy can induce significant toxicities, primarily cytokine release syndrome (CRS), characterized by systemic inflammation and flu-like symptoms (68, 69), and immune effector cell-associated neurotoxicity syndrome (ICANS), causing neurological manifestations such as disorientation, aphasia, and tremor (70). Additional adverse events include cytopenias and increased risk of infections (71, 72).

Since 2021, two second-generation anti-BCMA CAR-T therapies have been approved by the FDA and EMA for RRMM. Idecabtagene vicleucel (Ide-cel) and ciltacabtagene autoleucel (Cilta-cel) both use a 4-1BB co-stimulatory domain and lentiviral gene transfer. Pivotal clinical trials reported complete remission (CR) rates of 33–67%, with 87–95% experiencing cytokine release syndrome and 18–21% neurotoxicity (27, 28). Additionally, the Spanish Agency of Medicines (AEMPS) has authorized hospital-exempt use of the academic BCMA-directed CAR-T therapy ARI-0002h (cesnicabtagene autoleucel), developed at Hospital Clínic, Barcelona (73).

Although CLL was the first hematological disease treated with CAR-T cells, the responses to this immunotherapy have been characterized by inferior responses compared to other B-cell malignancies (74). In 2024, the FDA approved the first CAR-T therapy for rrCLL and SLL patients who have received at least 2 prior lines of therapy, including a BTKi and a BCL2 inhibitor. Lisocabtagene maraleucel (liso-cel) is an anti-CD19 CAR-T therapy featuring a 4-1BB co-stimulatory domain and lentiviral gene transfer, with a defined composition of CD4+ and CD8+ CAR-T cells. Clinical studies reported a CR rate of 18%, with treatment-related toxicities including CRS in 83% of patients and ICANS in 46% (75). Another recent study suggests that the third-generation CAR-T, HD-CAR-1, can induce prolonged complete responses in rrCLL patients (76). Therefore, although CAR-T cell therapy has recently entered the therapeutic landscape of rrCLL/SLL, the modest complete response rates and the persistence of relapse support the development of rational synergistic combinations to enhance CAR-T cell expansion, persistence, and antitumor efficacy. Although no CAR T-cell product is specifically approved for RT, commercial anti-CD19 CAR T-cell therapies—axicabtagene ciloleucel, tisagenlecleucel, or liso-cel are under evaluation (77, 78). In 2025, a multicenter analysis confirmed durable remissions in a subset of RT patients, although relapse remains a limitation especially in TP53-aberrant cases (79).

#### 1.2.3 Bispecific antibodies

Bispecific antibodies (BsAbs) are an emerging form of T-cell redirecting immunotherapy in B-cell malignancies. Structurally, BsAbs are engineered monoclonal antibodies capable of simultaneously binding two different epitopes: one on tumor-associated antigens expressed by malignant cells and the other on CD3 of T cells (80–82). This dual-binding forms an immunological synapse, activating both CD4+ helper and CD8+ cytotoxic T cells independently of MHC-mediated antigen presentation (80, 83). Activated T cells then mediate cytotoxicity primarily through the release of perforin and granzymes, as well as cytokine secretion, while BsAbs that retain an Fc region can also trigger additional immune effector mechanisms, including complement-dependent cytotoxicity (CDC), antibody-dependent cellular cytotoxicity (ADCC), and antibody-dependent cellular phagocytosis (ADCP) (84, 85). To date, 13 BsAbs have received regulatory approval across various cancer types, with over 100 molecules currently under clinical investigation (82, 84, 86). Unlike CAR-T cell therapies, which are typically administered as a single infusion, BsAbs require repeated and continuous dosing, often maintained until disease progression or unacceptable toxicity (87). Step-up dosing regimens, in which the therapeutic dose is gradually increased over several days, are commonly employed to mitigate immune-related adverse events such as CRS, ICANS or infusion-related reactions (88, 89).

Several BsAbs have been developed for the treatment of MM, targeting different tumor antigens. Anti-BCMA/CD3 BsAbs include teclistamab, elranatamab, linvoseltamab, ABBV-383, and alnuctamab (29, 90–92). BsAbs targeting GPRC5D/CD3 include talquetamab and forimtamig (30, 93), while cevostamab targets Fc receptor-homolog 5 (FcRH5) and CD3 (94). Currently, teclistamab, elranatamab, linvoseltamab and talquetamab are approved for relapsed or refractory MM. Clinical studies reported CR rates of 28–39%. Common adverse events include CRS (52–77%) and ICANS (3–15%) (29, 30, 90).

In the treatment of CLL, and particularly in the context of RT, the potential of anti-CD20/CD3 bispecific antibodies have been explored, although none has yet been approved. These agents include mosunetuzumab (95), epcoritamab, and glofitamab (96). In patients with RT, epcoritamab monotherapy showed clinically meaningful antitumor activity, with a safety profile consistent with previous studies, supporting further investigation in this setting (97).

Taken together, in both MM and CLL, it has been shown that although immunotherapy has expanded the therapeutic landscape of B-cell malignancies, its efficacy remains variable across diseases and clinical settings and is still limited by incomplete responses, relapses, toxicity and mechanisms of tumor immune escape. In this context, combination strategies are of particular interest, as they may enhance effector-cell function, improve target-cell susceptibility, overcome the immunosuppressive tumor microenvironment, and ultimately increase the depth and durability of response. Therefore, this review aims to summarize the current evidence on synergistic immunotherapeutic combinations in MM and CLL, including RT, with a particular focus on their biological rationale, clinical evidence, current limitations, and the emerging role of the gut microbiota in immunoregulation.

## 2 Combination of immunotherapies with targeted agents

### 2.1 Combinational treatments based on immunotherapies in MM

#### 2.1.1 Checkpoints inhibitors in combination therapies

Given the limited efficacy of ICIs as monotherapy in MM, multiple combination strategies have been explored to overcome the challenges found (98).

##### 2.1.1.1 Anti-PD-1–based combinations

Most clinical experience with ICIs in MM has been generated with anti-PD-1 antibodies, particularly pembrolizumab and nivolumab, in combination with standard antimyeloma therapies. Among them, IMiDs provided the earliest and strongest mechanistic rationale for combination with PD-1 blockade. These agents have been shown to increase interferon signalling and enhance cytotoxic T- and NK-cell activity, and reduce regulatory T cells (Tregs), thereby potentially counteracting T-cell dysfunction in MM (99). Early clinical signals were observed in the phase I KEYNOTE-023 trial (NCT02036502), in which pembrolizumab combined with lenalidomide–dexamethasone achieved an overall response rate (ORR) of 76% in RRMM patients with relatively low rates of immune-mediated toxicity (100). However, this efficacy did not translate into clinical benefit in randomized phase III testing. In KEYNOTE-183 (NCT02576977), comparing pembrolizumab plus pomalidomide–dexamethasone with pomalidomide–dexamethasone alone in RRMM, the triple combination resulted in inferior progression-free survival (PFS) (median 5.6 vs 8.4 months), a trend towards worse overall survival (OS), higher rates of grade 3–5 toxicity (83% vs 65%) and more deaths (29 vs 21) (101). Similarly, in KEYNOTE-185 (NCT02579863), the addition of pembrolizumab to lenalidomide-dexamethasone in NDMM patients showed no PFS improvement (6-month PFS 82% vs 85%) and increased serious adverse events (54% vs 39%) and more treatment-related deaths (4% vs 1%) (102). These trials were prematurely terminated, establishing that systemic PD-1 blockade combined with IMiDs leads to clinically meaningful immune toxicity without a survival benefit in unselected MM populations. Mechanistically, the failure of combinations of pembrolizumab and IMiDs suggests that PD-1/PD-L1 is only one component of the complex immunosuppressive network operating in MM. This network also involves chronic antigen exposure, suppressive myeloid cells, stromal support, TGF-β signalling, and additional inhibitory receptors. Therefore, PD-1 blockade alone may be insufficient to restore effective antimyeloma immunity, while the addition of IMiDs may enhance systemic immune activation and toxicity without adequately rescuing tumour-specific T-cell function, particularly in unselected patient populations (103).

Proteasome inhibitors (PIs) have also been investigated as partners for PD-1 blockade based on their capacity to induce immunogenic cell stress, enhance antigen presentation, and reshape myeloid inflammation within the tumour microenvironment (TME) (98). Despite strong preclinical data suggesting that carfilzomib could sensitize tumours to PD-1 inhibition (104), clinical results have been disappointing. In cohort 2 of the phase I KEYNOTE-023 trial, pembrolizumab with carfilzomib-dexamethasone in RRMM patients, resulted in modest PFS and OS (14.3 and 22.5 months, respectively); consequently, the strategy did not advance further (105). This discrepancy suggests another biological limitation: the release of antigen or visibility is unlikely to be sufficient if effector T cells remain functionally exhausted, and the marrow niche remains non-permissive. From a clinical-design perspective, PI-based backbones may also provide insufficient immune priming themselves to generate a meaningful synergistic effect with PD-1 blockade. Consequently, current strategies are focused on preparing the tumour microenvironment, by increasing antigen visibility and inflammatory signalling, either before or alongside PD-1 inhibition. In this context, the AMBUSH trial (NCT05514990) and NCT03605719 are evaluating the oncolytic virus pelareorep combined with PIs and ICIs. The aim of these studies is to test if enhancing innate immune activation and antigen presentation can convert PI backbones into more ICI-responsive platforms. However, the efficacy of these methods remains to be fully elucidated (106).

Monoclonal antibodies such as elotuzumab (SLAMF7) and daratumumab(CD38) exert antimyeloma activity through ADCC, mediated by both NK and T cells and negatively regulated by the PD-1/PD-L1 axis (107, 108). Preclinical studies suggested that PD-1 blockade could enhance elotuzumab-driven immune effector function (109). Nevertheless, clinical translation has been unsuccessful. In the phase III CheckMate 602 trial (NCT02726581), the addition of nivolumab to pomalidomide–dexamethasone, with or without elotuzumab, did not improve PFS or OS and was associated with increased toxicity, resulting in discontinuation of this strategy (110). It is evident that the probable biological constraint is that enhancing a solitary effector mechanism is incapable of surmounting the multifaceted immune evasion architecture of MM.

Overall, these data suggest that successful ICI-based combinations in MM will likely require not only rational partners, but also a permissive immune context, biomarker-guided stratification, and strategies capable of addressing multiple mechanisms of immune resistance simultaneously.

##### 2.1.1.2 Anti-PD-L1–based combinations

Experience with anti-PD-L1 antibodies in MM has been more limited and similarly disappointing. Although strong preclinical rationale supported targeting the PD-1/PD-L1 axis to enhance daratumumab-mediated immune responses in MM (46), this rationale may have been biologically incomplete. Resistance to CD38-directed therapy in MM is multifactorial and involves not only T-cell inhibition, but also reduced CD38 expression, upregulation of complement inhibitory proteins, effector-cell dysfunction, and persistence of an immunosuppressive bone marrow microenvironment. In this context, it appears that PD-L1 blockade alone is unlikely to fully restore sensitivity to daratumumab in cases of refractory disease (111, 112).

In line with this, multiple early-phase clinical trials evaluating daratumumab in combination with PD-1 or PD-L1 inhibitors in RRMM (e.g., NCT02431208, NCT01592370, NCT03357952, NCT03000452) failed to demonstrate a favourable risk–benefit profile. Notably, in the phase II study NCT03000452, which evaluated the combination of daratumumab and the PD-L1 inhibitor durvalumab in patients with RRMM who had failed to respond to daratumumab, it was observed that among the 18 patients who received the treatment, none achieved a partial response or better. Furthermore, the study noted that serious adverse events occurred in 38.9% of patients, indicating that checkpoint blockade alone is insufficient to overcome resistance to CD38-directed therapies (46).

##### 2.1.1.3 Other emerging treatments

Emerging immunomodulatory strategies aim to reshape the immune context in MM to enhance responsiveness to immunotherapy. Among these, activation of the stimulator of interferon genes (STING) pathway has emerged as a promising approach, as it plays a central role in linking innate and adaptive immunity by inducing type I interferons, promoting antigen presentation, and priming CD8+ T cells, ultimately leading to durable antitumor immune responses (113, 114). It is frequently observed that resistance to ICIs is associated with an absence of a pre-existing antitumor immune response (115). Clinical data from other hematologic malignancies indicates that the activation of the STING pathway combined with checkpoint inhibition may trigger a more effective antitumor immune response (114, 116, 117). In the specific context of MM, preclinical studies have demonstrated that the combination of STING activation with PIs and PD-1 blockade results in a survival advantage (114). Concurrently, DNA vaccines constitute an alternative strategy that aims to induce *de novo* tumor-specific immunity. Notwithstanding the fact that DNA vaccines alone frequently have poor immunogenicity, preclinical studies in murine MM models using a GPRC5D-targeted DNA vaccine have demonstrated that this is significantly amplified by PD-1 blockade (118).

#### 2.1.2 CAR-T cells in combination therapies

Despite their clinical success, CAR-T cell therapies in MM face important limitations, including relapse, limited persistence, and treatment-related toxicities. Combining CAR-T cells with standard therapies offers a rational strategy to overcome these challenges (119, 120).

##### 2.1.2.1 BCMA-directed CAR-T cells

Idecabtagene vicleucel (ide-cel) was the first BCMA-directed CAR-T therapy approved for RRMM. Although ide-cel induces high initial response rates, relapse remains frequent, providing a strong rationale for combinatorial approaches. IMiDs represent the most extensively studied partners for ide-cel. As demonstrated by preclinical data lenalidomide and pomalidomide have shown to enhance T-cell activation, increase IL-2 production, augment NK and CD8^+^ cytotoxicity, and reduce regulatory T cells (121–123). In MM models, lenalidomide has been demonstrated to enhance persistence, immune synapse formation, and Th1 cytokine secretion in CS1/SLAMF7-directed CAR-T cells (124) and to enhance activation, proliferation, and cytokine release of BCMA-directed CAR-T cells (125). These findings support the idea that IMiDs may partially overcome one of the central limitations of CAR-T therapy in MM, namely the inadequate expansion and functional persistence.

The first clinical evidence was reported in a case of RRMM in which lenalidomide administered prior to a second BCMA CAR-T infusion resulted in rapid CAR-T expansion and a very good partial response (VGPR) within 14 days, outperforming delayed administration (126). A retrospective study further showed that pomalidomide maintenance after BCMA CAR-T significantly prolonged median time to progression (13 months vs. 5.85 months) and OS (not reached vs. 10.7 months), without long-term hematologic or hepatic toxicity (127). A similar benefit was observed in patients with extramedullary disease, a population typically associated with inferior CAR-T outcomes (128). In earlier disease setting, a pilot study evaluating sequential anti-CD19 and anti-BCMA CAR-T cell therapy followed by lenalidomide maintenance after ASCT reported sustained minimal residual disease (MRD) negativity in 8 of 10 patients, with all patients achieving CR or VGPR and no relapses observed after 18 months (129). Collectively, these data suggest that IMiD-based maintenance may be particularly useful when relapse is driven by limited CAR-T persistence rather than complete loss of BCMA expression. Multiple ongoing studies are expected to refine the role of IMiD-based strategies as peri- and post-CAR-T partners.

Prospective validation is ongoing. The BMT CTN 1902 trial (NCT05032820) is evaluating the efficacy of ide-cel followed by lenalidomide maintenance in patients who have achieved MRD-positive or suboptimal responses following ASCT. Beyond conventional IMiDs, CRBN E3 ligase modulators (CELMoDs), iberdomide (CC-220) and mezigdomide (CC-92480), have been designed as promising and more selective agents (130). Recent preclinical studies have indicated that iberdomide promotes increased expansion and cytotoxic function of anti-BCMA CAR-T cells *in vitro* (131). The multi-arm phase I/II KarMMa-7 study (NCT04855136) is assessing ide-cel in combination with iberdomide, a non-IMiD partner (BMS-986405), or IMiD-based triplets delivered before, during, and/or after CAR-T infusion. Mature efficacy data are not yet available (132). In contrast, KarMMa-9 (NCT06045806) designed to evaluate ide-cel plus lenalidomide maintenance in NDMM, was discontinued due to the challenges of integrating CAR-T cell therapy into early treatment settings, resulting in fewer eligible patients. This demonstrates that certain combination failures may be indicative of clinical feasibility and trial design limitations, as opposed to a paucity of biological rationale alone.

Ciltacabtagene autoleucel is a second-generation BCMA CAR-T associated with deep and durable responses, that is also being incorporated in clinical trials of combination IMID-CAR-T. CARTITUDE-2 cohort D demonstrated that cilta-cel followed by lenalidomide maintenance in NDMM patients with suboptimal post-ASCT response achieved CR rates of 94%, MRD negativity of 80%, and 18-month PFS and OS of 94%, with manageable toxicity (133, 134). Whilst these results are encouraging, they must be interpreted with caution until they are confirmed in larger studies and in relation to underlying resistance biology, including BCMA modulation and CAR-T-cell persistence.

Beyond IMiD- and CELMoD-based strategies, alternative combination approaches are now being explored to enhance CAR-T cell fitness and tumor susceptibility by modulating the tumor microenvironment and intracellular signalling pathways. Selinexor, a selective oral XPO1 inhibitor approved in combination with dexamethasone for RRMM, has emerged as a potential immunomodulatory partner for CAR T-cell therapy. As demonstrated in preclinical studies, XPO1 inhibition has been shown to enhance the activation of both CD8+ T-cells and NK-activation, while reducing suppressive myeloid populations (136, 137). Furthermore, low dose selixenor has been demonstrated to upregulate BCMA expression *in vitro*, which may increase target density and facilitate CAR-T recognition of myeloma cells (138). A retrospective analysis demonstrated deep and durable responses when selinexor was administered prior to CAR-T infusion (139). Data from the US Myeloma Immunotherapy Consortium showed that patients with RRMM receiving selinexor-based bridging before ide-cel achieved a median PFS of 9.8 months, comparable to IMiD-based bridging strategies (140). In a multicentre retrospective analysis of heavily pretreated RRMM patients reported prior exposure to selinexor did not compromise outcomes following BCMA-directed CAR-T cell therapy (141). Selinexor use immediately prior to CAR-T was associated with a trend toward reduced risk of progression and mortality (142, 143). Prospective validation is ongoing in the phase I trial NCT05201118, which is evaluating selinexor in combination with CT103A, a fully human BCMA-directed CAR-T product designed to reduce immunogenicity and improve persistence, focusing on safety, pharmacokinetics, pharmacodynamics, and immune correlates.

Mechanistically, the appeal of selinexor as a partner lies in its potential to act on both sides of the interaction: it may improve CAR-T-cell fitness while simultaneously increasing myeloma-cell vulnerability. However, the current evidence remains largely retrospective or preclinical, and it is still unclear whether its main benefit comes from better disease control during bridging, improved antigen density, modulation of the immune microenvironment, or a combination of these effects (135, 136, 144).

##### 2.1.2.2 GPRC5D-directed CAR-T cells

To overcome BCMA antigen escape, GPRC5D-directed CAR-T therapies are being explored. The ongoing study NCT06121843 is evaluating arlocabtagene autoleucel (BMS-986393) in combination with alnuctamab, iberdomide, or mezigdomide, reflecting a broader strategy to integrate CELMoDs with next-generation CAR-T platforms.

#### 2.1.3 Bispecific antibodies in combination therapies

BsAbs have emerged as a promising option by mediating direct cytotoxic interactions between T cells and myeloma cells especially in relapsed or refractory MM. However, they have presented several limitations associated with toxicities, T-cell exhaustion, or limited durability of responses due to the development of resistance or antigen escape. These challenges highlight the need to optimize their therapeutic potential. Consequently, increasing attention has been directed toward combination therapeutic strategies.

##### 2.1.3.1 BCMAxCD3 BsAbs

Several BCMAxCD3 BsAbs have been evaluated in MM patients. The first BsAb was Pacanalotamab (AMG-420) which has been administered intravenously as Pavurutumab (AMG-701) in MM (145, 146). Despite the results of both in combination with IMiDs, showing high efficacy in *in vivo* models (147), clinical trials to test them in combination with pomalidomide and dexamethasone were terminated due to business reasons, since other bispecific antibodies were developed to be administrated subcutaneously.

Teclistamab (Tec) is a BCMAxCD3 BsAb approved in 2022 based on the results of MajesTEC-1 trial (90). Currently, there are further on-going clinical trials that are evaluating the efficacy of Tec in combination therapies. Among them, the MajesTEC-2 trial is an active multi-arm phase 1b trial carried out to characterize the safety and tolerability of Tec when administered in different combination regimens and to identify its optimal dose in these combinations. For this reason, this study includes several cohorts in which a different combination is assessed (Cohort A: Tec + Daratumumab (D) + Pomalidomide (P), B: Tec + D + Lenalidomide (R) + Bortezomib (V) (21 day cycle), C: Tec + nirogacestat, D: Tec + R, E: Tec + D + R, F: Tec + D + R + V (28 day cycle)). In this setting, the drugs used as adjuvants, might enhance the BsAb efficiency when used as monotherapy, through immunomodulation. For instance daratumumab may support Tec by depleting CD38+ immune-regulatory cells and promoting T-cell expansion (148), the IMiDs lenalidomide and pomalidomide can reinforce both NK and T-cell activity (149, 150), moreover lenalidomide improves T-cell fitness (151), nirogacestat can increase membrane BCMA in both *in vitro* and ex vivo conditions (152) by blocking γ-secretase-mediated shedding (153) and bortezomib can increase tumor susceptibility through immunogenic stress (154) besides promoting immunogenic cell death (155).

Preliminary results of Tec-DP achieved an ORR of 88.5% and a CR of 61.5% (143). Interestingly, Tec + nirogacestat showed an ORR and CR of 77.8% and 51.9%, respectively, in RRMM (156). In addition, Tec-R resulted in an ORR of 74.2% and a CR or better (≥CR) of 35.5% in RRMM patients, further leading to phase 3 MajesTEC-4 of NDMM patients (136). Also, Tec-DR obtained ORR of 93.5% with a CR of 54.8% in RRMM patients, leading to the MajesTEC-7 trial to assess this combination in NDMM patients (157).

Another trial is the phase 3 MajesTEC-3 trial, which compared the efficacy of the combination of Tec-D with daratumumab along with either pomalidomide and dexamethasone or bortezomib and dexamethasone, being the same rationale as before, were daratumumab could deplete CD38+ immune regulatory cells and promote T-cell expansion, while the IMiD or proteasome inhibitor may further potentiate the activity of Tec. The study showed that the combination Tec-D compared to the patients who did not receive the BsAb increased the ORR (89.0% vs 75.3%) and remarkably the CR (81.8% vs. 32.1%), respectively (158). Also, Tec-D could be a viable option in NDMM elderly patients, as shown in the phase 2 IFM2021–01 trial, where all the patients responded to the treatment with a VGPR or better (≥VGPR) of 97%, while being well-tolerated (159). The phase 3 MajesTEC-4 also assessed the efficacy of the combination of Tec, either as monotherapy or along with lenalidomide (teclistamab weekly or every 4 weeks) in NDMM patients and its preliminary results showed that a MRD and a CR of 100% was achieved in the combination cohorts meaning that it could be a viable option post-transplant in NDMM. As before, lenalidomide, could complement Tec by enhancing myeloma-specific T-cell responses and amplifying anti-myeloma cellular immunity. In addition, derived from the MajesTEC-2 trial, there is the phase 3 MajesTEC-7 trial, an on-going clinical trial in which the effects of Tec-DR versus the combination DRd are being evaluated in patients with NDMM patients who are not legible or intended to an ASCT. Safety results from this trial showed an ORR of 92, 3% and a CR of 73, 1% in a small cohort (160, 161). Also, some results from the phase 2 MajesTEC-5 study, that assessed Tec-based combination regimens in transplant-eligible NDMM patients, had showed the preliminary results of various cohorts (A: Tec(Weekly)-DR, A1: Tec(Every 4 weeks)-DR, B: Tec-DVR) achieving MRD negativity and an ORR in all the patients along with a CR of 100% in cohorts A and A1 and 73, 7% in cohort B, maintaining its feasibility. Again, the mechanistic rationale keeps the same background, Tec redirects T cells, while daratumumab can reduce CD38+ suppressive populations, lenalidomide can amplify T/NK-cell activity, and bortezomib may add proteotoxic stress that increases tumor susceptibility. Therefore, this combinational regimen could be a clear manageable option for TE-NDMM patients. The latest of these trials, the phase 3 MajesTEC-9 trial is being carried out and thus, results have not been presented yet, and it will compare the efficiency between Tec plus pomalidomide, bortezomib and dexamethasone or plus carfilzomib and dexamethasone in RRMM patients with 1–3 prior lines of therapy.

Another BCMA-CD3 BsAb, Elranatamab (Elra), was approved for RRMM in 2023. Related to Elra in combination therapies, results from the first part of the phase 3 MagnetisMM-6 clinical trial has been published (162), firstly stablishing the optimal dose of the combination of Elra with lenalidomide alone or with daratumumab and then making a comparison with the combination DRd in RRMM or transplant-ineligible NDMM patients. As in Tec-based combinations, the rationale for combining Elra with daratumumab and lenalidomide is similar. Preliminary results about efficacy had been confirmed as of data cut-off (DCO) with an ORR of 91, 9% having 81, 1% of the patients a VGPR or better, and in patients enrolled ≥4 months before DCO, the ORR was 95, 7% achieving all patients a VGPR (163). In addition, the first part of the phase 1 MagnetisMM-20 study (164), that had assessed the combination of Elra with carfilzomib and dexamethasone which, demonstrated clinical efficacy of this combination with a non-confirmed ORR of 100% and a confirmed ORR of 83.3% (164). In this setting, carfilzomib may provide additive cytotoxic pressure through proteasome inhibition and proteotoxic stress (165) and similar to bortezomib, by induction of immunogenic cell death (166) potentially complementing the T-cell redirecting activity of Elra. Moreover, the combination of Elra with iberdomide, was assessed in phase 1 MagnetisMM-30 study (167), confirming its safety and efficacy as it had shown a confirmed ORR of 77, 3% with and 45, 5% of patients had CR of better and 68, 2% VGPR or better (168). In this case, iberdomide might be mechanistically attractive because it has been shown to increase effector T and NK cells subsets in MM, which may reinforce the immune synapse created by Elra (169). There are some many on-going MagnetisMM clinical trials enrolling RRMM patients to assess different therapies, but no results have been published yet. The phase 1B/2 MagnetisMM-4 umbrella study (NCT05090566) is a clinical trial which will assess the combination of Elra with nirogacestat or with lenalidomide paired with dexamethasone (170). Also, the MagnetisMM-5 phase 3 clinical trial (NCT05020236) will be composed of two parts to assess safety and activity of the combination therapy. In the first, patients will receive different doses of Elra-D and in the second, patients will receive Elra-D or Elra-DPd (171).

Linvoseltamab is the most recently approved BCMAxCD3 BsAb. Linvoseltamab is assessed in combination with carfilzomib in on-going the phase 1b LINKER-MM2 trial (172) and preliminary results have been shown, achieving an ORR of 91% when the dose of linvoseltamab was 100mg and an ORR of 100% when it was 150mg, also the PFS at 6 and 12 months were 91% and 73% respectively for the 100mg dose. The rationale for this combination is similar as the one used in Elra combination, as carfilzomib may provide additive cytotoxic pressure through proteasome inhibition, potentially complementing the T-cell–redirecting activity of linvoseltamab.

##### 2.1.3.2 GPRC5DxCD3 BsAbs

Talquetamab (Tal) is a T-cell redirecting bispecific antibody targeting GPRC5D. Its efficacy in combination with pomalidomide for RRMM patients is being assessed in the on-going phase 1b MonumenTAL-2 study (173), achieving high rates of response and complete remission (60%) when Tal was dosed weekly rather than every other week. Also, one cohort of the TRIMM-2 trial assessed Tal-DP, and similarly to the previous trial, when dosed weekly, the response was total and the CR rate was high too (56%). However, when Tal is combined with daratumumab only, higher response rates were higher when dosed every other week, although CR and PFS rates remain similar (174, 175). Furthermore, the phase 3 MonumeTAL-3 study is being carried out, comparing the efficacy of Tal-DP or Tal-D combinations with DPd. However, results have not yet been published in (176). From a mechanistic perspective, similarly to BCMAxCD3 BsAbs-based combinations, both pomalidomide and daratumumab may complement Tal efficacy through T-cell expansion and reduction on CD38+ immunoregulatory populations.

Alternatively, Tal is being assessed with other immunotherapies. In the on-going phase 2 aMMbition clinical trial, cilta-cel, Tal and Tec are being evaluated while combined with daratumumab, following an induction with DVRd in high-risk NDMM. However, due to the recent set of this clinical trial, results have not been published yet. Then, there is the phase 1 TRIMM-3 study, where the safety and efficacy of the combination of Tal and a PD-1 inhibitor (cetrelimab) is being evaluated in RRMM patients. PD-1 blockade may help to preserve effector T-cell function during BsAb therapy by avoiding T-cell exhaustion. Preliminary results showed an ORR of 70, 5% and ≥CR was achieved in 40, 9% of patients without prior therapies, while in patients with prior BsAb therapy, efficacy results were quite lower. Interestingly, the on-going phase 1b/2 RedirecTT-1 clinical study is evaluating the combination of Tal and Tec in RRMM patiens including those with extramedullary disease. Its main results were an ORR of 79-80% and CR of 53-61% considering both phases. Thus, being as effective as the previously mentioned combination therapies. However, grade 5 adverse events were observed in 12, 2% of the patients (177, 178).

Overall, accumulating evidence demonstrates that bispecific antibody-based combination therapies targeting BCMA (Teclistamab, Elranatamab, Linvoseltamab) or GPRC5D (Talquetamab) have shown encouraging clinical efficacy in MM across different disease settings. Compared with monotherapy and standard regimens, these combinations consistently achieve markedly higher overall response and CR rates, including deep responses such as MRD negativity, not only in heavily pretreated relapsed/refractory patients but also in newly diagnosed, transplant-eligible and ineligible populations. The incorporation of IMiDs, anti-CD38 antibodies, proteasome inhibitors, or even dual bispecific approaches appears to potentiate T-cell mediated cytotoxicity and improve response depth and durability, suggesting a potential paradigm shift toward earlier use of these agents. However, this increased efficacy is accompanied by a higher incidence of grade 3/4 cytopenias and infections, underscoring the importance of optimized dosing schedules, patient selection, and proactive toxicity management. Also, the evidence is still dominated by early-phase trials, non-randomized cohorts and heterogeneous patient populations, which limits firm comparisons across regimens and disease settings. Ongoing phase 3 trials will be crucial to define optimal combinatorial strategies and to establish the long-term balance between efficacy, safety, and treatment sustainability in multiple myeloma.

**Figure 1:**
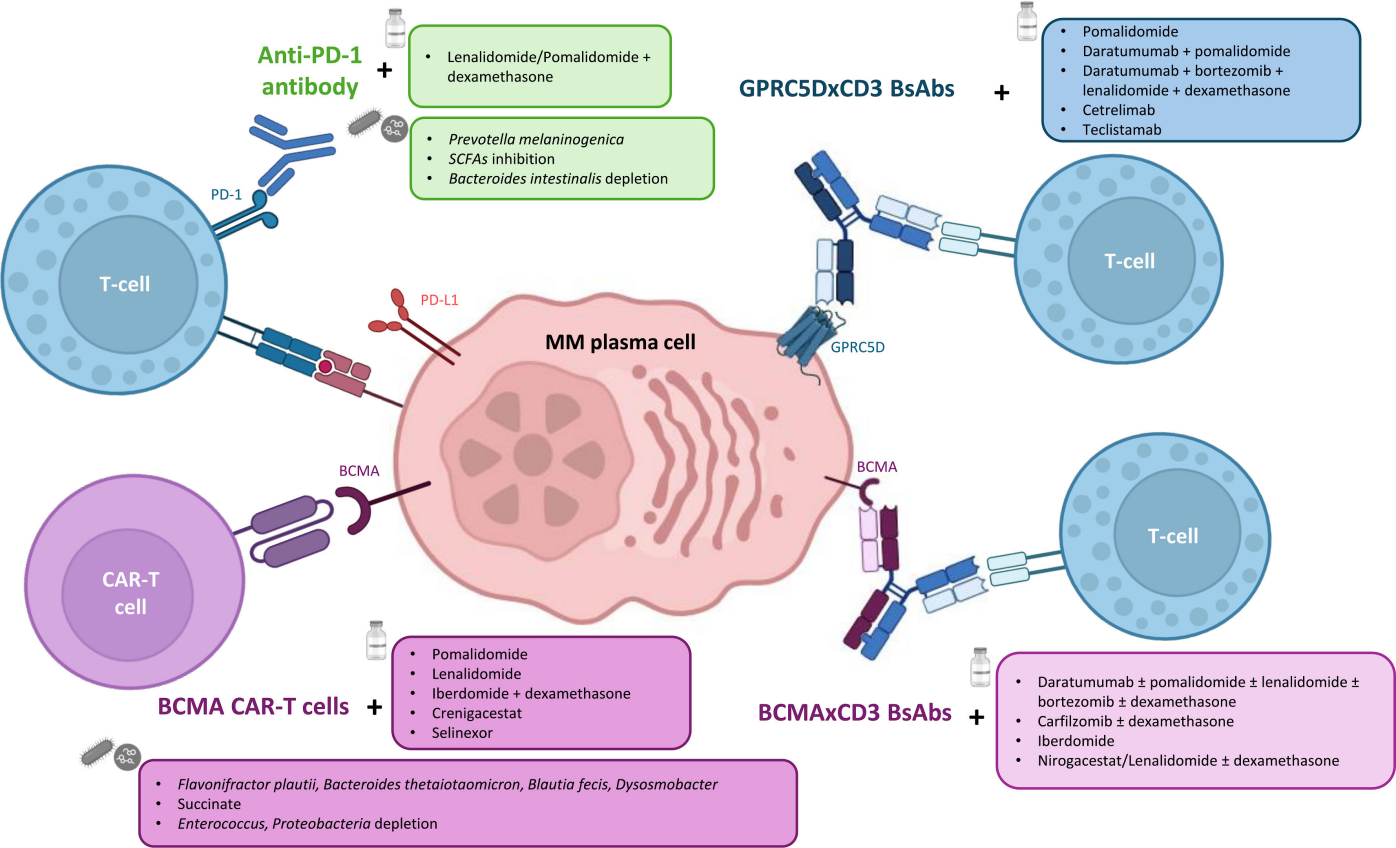
Immunotherapy in combinatorial therapies in MM and effects of gut microbiota in these therapies.

As a summary, **Figure 1** includes each combinational regimen described in MM, with the main therapies and the drugs that they are combined with and an overview of the clinical trials discussed in this review is provided in **Table 1**.

**Table 1:** Clinical trial assessing combinational treatments based on immunotherapies for the treatment of MM, CLL and RT.

Disease	Clinical trial	Phase	Immunotherapy	Drug (target)	Main improvements	Ref.
RRMM	ChiCTR2000036350 (www.chictr.org.cn)	0	BCMA CAR-T cell therapy	Pomalidomide (CRBN)	ORR: 100%, CR: 100%	(29)
NDMM	NCT03455972	1/2	BCMA CAR-T cell therapy	Lenalidomide (CRBN)	ORR: 100%, CR: 100%, MRD negativity(42m): 70%	(129)
NDMM	NCT04133636 (CARTITUDE-2) (Cohort D)	2	Ciltacabtagene autoleucel (BCMA CAR-T cell)	Lenalidomide (CRBN)	ORR: 94.1%, MRD negativity: 80%, PFS(18m): 93.8%, OS(18m): 93.8%	(133, 134)
MM	NCT05032820	2	Idecabtagene vicleucel (BCMA CAR-T cell)	Lenalidomide (CRBN)	ORR: Not specified, CR: 63%, MRD negativity: 87%	
RRMM	NCT04855136 (KarMMa-7)	1/2	Idecabtagene vicleucel (BCMA CAR-T cell)	i- Iberdomide (CRBN) + dexamethasone ii-BMS-986405 (γ-secretase)	No results yet	(132)
RRMM	NCT03502577	1	BCMA CAR-T cell therapy	Crenigacestat (γ-secretase)	ORR: 89%, CR: 55%	(179)
RRMM	NCT05201118	1	BCMA CAR-T cell therapy	Selinexor (XPO1)	No results yet	
RRMM	NCT02036502 (KEYNOTE-023)	1	Pembrolizumab (anti PD-1)	Lenalidomide (CRBN) + dexamethasone	ORR: 44%, CR: 4%	(100)
RRMM	NCT02576977 (terminated)	3	Pembrolizumab (anti PD-1)	Pomalidomide (CRBN) + dexamethasone	PFS: 5.6 months, OS: Not reached	(101)
NDMM	NCT02579863 (terminated)	3	Pembrolizumab (anti PD-1)	Lenalidomide (CRBN) + dexamethasone	PFS: Not reached	(102)
RRMM	NCT02036502	1	Pembrolizumab (anti PD-1)	Carfilzomib (Proteasome 26S) + dexamethasone	ORR: 70%, CR: 0%, PFS: 14.3 months, OS: 22.5 months	(105)
RRMM	NCT04722146 (MajesTEC-2 Cohort A)	3	Teclistamab (BCMAxCD3 BsAbs)	Daratumumab (CD38) + pomalidomide (CRBN)	ORR: 88.5%, ≥CR:61.5%, ≥VGPR: 84.6%, median PFS: 26.5mo	(143)
RRMM	NCT04722146 (MajesTEC-2 Cohort C)	3	Teclistamab (BCMAxCD3 BsAbs)	Nirogacestat (γ-secretase)	ORR: 77.8%, CR:51.9%	(156)
RRMM	NCT04722146 (MajesTEC-2 Cohort D)	3	Teclistamab (BCMAxCD3 BsAbs)	Lenalidomide (CRBN)	ORR: 74.2%, ≥CR:35.5%	(136)
RRMM	NCT04722146 (MajesTEC-2 Cohort E)	3	Teclistamab (BCMAxCD3 BsAbs)	Daratumumab (CD38) + lenalidomide (CRBN)	ORR: 93.5%, ≥CR:54.8%, ≥VGPR: 90.3	(157)
RRMM	NCT05083169 (MajesTEC-3)	3	Teclistamab (BCMAxCD3 BsAbs)	Daratumumab (CD38)	ORR: 89.0%, ≥CR:81.8%	(158)
NDMM	NCT05572229 (IFM2021-01)	2	Teclistamab (BCMAxCD3 BsAbs)	Daratumumab (CD38)	ORR: 100%, ≥VGPR:97%	(159)
NDMM	NCT05243797 (MajesTEC-4 Cohort 1)	3	Teclistamab (BCMAxCD3 BsAbs)	Lenalidomide (CRBN)	ORR: Not specified, ≥CR:100%, MRD negativity: 100%	
NDMM	NCT05552222 (MajesTEC-7)	3	Teclistamab (BCMAxCD3 BsAbs)	Daratumumab (CD38) + lenalidomide (CRBN)	ORR: 92.3%, ≥CR:73.1%, ≥VGPR: 92.3%	(161)
NDMM	NCT05695508 (MajesTEC-5)	2	Teclistamab (BCMAxCD3 BsAbs)	Daratumumab (CD38) + lenalidomide (CRBN) ± bortezomib (Proteasome 26S)	ORR: 100%, ≥CR(Cohort A): 100%, ≥CR(Cohort B): 73.7%	
RRMM	NCT05572515 (MajesTEC-9)	3	Teclistamab (BCMAxCD3 BsAbs)	Pomalidomide (CRBN) + bortezomib (Proteasome 26S) + dexamethasone	No results yet	(180)
Transplant ineligible-NDMM	NCT05623020 (MagnetisMM-6)	3	Elranatamab (BCMAxCD3 BsAbs)	Daratumumab (CD38) + lenalidomide (CRBN)	ORR: 91.9%, ≥VGPR:81.8%	(163)
RRMM	NCT05675449 (MagnetisMM-20)	1	Elranatamab (BCMAxCD3 BsAbs)	Carfilzomib (Proteasome 26S) + dexamethasone	ORR: 83.3%	(164)
RRMM	NCT06215118 (MagnetisMM-30)	1	Elranatamab (BCMAxCD3 BsAbs)	Iberdomide (CRBN)	ORR: 77.3%, ≥CR:45.5%, ≥VGPR: 68.2%	(168)
RRMM	NCT05090566 (MagnetisMM-4)	1b/2	Elranatamab (BCMAxCD3 BsAbs)	Nirogacestat (γ-secretase) or lenalidomide (CRBN) + dexamethasone	No results yet	(176)
RRMM	NCT05020236 (MagnetisMM-5)	3	Elranatamab (BCMAxCD3 BsAbs)	Daratumumab (CD38) ± (Pomalidomide (CRBN) + dexamethasone)	No results yet	(171)
RRMM	NCT05137054 (LinkerMM-2)	1b	Linvoseltamab (BCMAxCD3 BsAbs)	Carfilzomib (Proteasome 26S)	ORR(100mg Linvo):100%, PFS(6mo): 91%, PFS(1y): 73%	(172)
NDMM	NCT05050097 (MonumenTAL-2)	1b	Talquetamab (GPRC5DxCD3 BsAbs)	Pomalidomide (CRBN)	ORR: 86.7%, ≥CR: 60.0% (QW cohort), ORR: 83.3%,≥CR: 44.5% (Q2W cohort)	
RRMM	NCT04108195 (TRIMM-2) (Tal-DP cohort)	2	Talquetamab (GPRC5DxCD3 BsAbs)	Daratumumab (CD38) + pomalidomide (CRBN)	ORR: 100%, ≥CR: 55.6% (QW cohort), ORR: 76.3%,≥CR: 55.9% (Q2W cohort)	
RRMM	NCT04108195 (TRIMM-2) (Tal-D cohort)	2	Talquetamab (GPRC5DxCD3 BsAbs)	Daratumumab (CD38)	ORR: 71.4% (QW cohort), ORR: 82.4% (Q2W cohort)	(174)
RRMM	NCT05455320 (MonumenTAL-3)	3	Talquetamab (GPRC5DxCD3 BsAbs)	Daratumumab (CD38) ± pomalidomide (CRBN)	No results yet	(176)
NDMM	NCT06577025 (aMMbition)	2	Talquetamab (GPRC5DxCD3 BsAbs) + Teclistamab (BCMAxCD3 BsAbs) + Ciltacabtagene autoleucel (BCMA CAR-T cell)	Daratumumab (CD38) + bortezomib (Proteasome 26S) + lenalidomide (CRBN) + dexamethasone	No results yet	
RRMM	NCT05338775 (TRIMM-3)	1	Talquetamab (GPRC5DxCD3 BsAbs)	Cetrelimab (Anti-PD-1)	ORR: 70.5%, ≥CR:40.9% (Naive patients) ORR: 68.4%, ≥CR: 31.6% (Non-naive patients)	
RRMM	NCT04586426 (RedirecTT-1)	1b/2	Talquetamab (GPRC5DxCD3 BsAbs)	Teclistamab	ORR: 79-80%, CR: 53-61%	(178)
CLL/SLL	NCT04082897 (MOLTO)	2	Atezolizumab (anti-PD-L1)	Venetoclax (BCL-2) + obinutuzumab (CD20)	ORR: 67.9%, CR: 28.6%, PFS(1y): 42.9%, OS(1y): 64.3%	(181)
RT	NCT03321643	1	Atezolizumab (anti-PD-L1)	Rituximab (CD20) + gemcitabine + oxiplatin	ORR: 22%, CR: 11% (RT cohort)	(182)
CLL/SLL/RT	NCT02329847	1/2a	Nivolumab (anti-PD-1)	Ibrutinib (BTK)	ORR: 61%, CR: 0% (CLL cohort) ORR: 65%, CR: 10% (RT cohort)	(183)
RT	NCT02420912	2	Nivolumab (anti-PD-1)	Ibrutinib (BTK)	ORR: 42%, CMR: 80%, PMR: 20% (RT cohort)	(184)
RT	NCT03884998	1	Nivolumab (anti-PD-1)	Copanlisib (PI3K)	ORR: 31%, CR: 15% (RT cohort)	(185)
RT	NCT04271956 (RT-1)	2	Tislelizumab (anti-PD-1)	Zanubrutinib (BTK)	ORR: 58.3%, CR: 18.8% (RT cohort)	(186)
RT	NCT02343120	1/2	Tislelizumab (anti-PD-1)	Zanubrutinib (BTK)	ORR: 61.5%, CR:15.4%	(187)
rrCLL, RT RRMM	NCT02684617 (KEYNOTE-155)	1	Pembrolizumab (anti-PD-1)	dinaciclib (CDK9)	ORR: 29.4%, CR: 0% (CLL cohort) ORR: 21.1%, CR:10.4% (RT cohort)	(188)
rrCLL	NCT02332980	2	Pembrolizumab (anti-PD-1)	Ibrutinib (BTK)	N/A	(189)
CLL	NCT02640209	2	CAR-T cell therapy	Ibrutinib (BTK)	ORR: Not evaluated CR: 44%, PFS(4y): 70%, eOS(4y): 84%	(190)
rrCLL	NCT01865617	1/2	CAR-T cell therapy	Ibrutinib (BTK)	ORR: 83%, CR: 22%, PFS(1y):38%, OS(1y):64%	(191)
RT	NCT06043674	2	Glofitamab (CD20xCD3 BsAbs)	Polatumuzmab vedotin (CD79b), pirtobruitib (BTK) or atezolizumab (anti PD-L1)	No results yet	
RT	NCT06735664	1	Odronextamab (CD20xCD3 BsAbs)	Zanubrutinib (BTK)	No results yet	
CLL/SLL	NCT07218510 (LonGEVity)	2	Epcoritamab (CD20xCD3 BsAbs)	Venetoclax (BCL-2) + Obinutuzumab (CD20)	No results yet	

#### 2.1.4 Other potential combinations in preclinical models

Beyond IMiDs and CELMoDs, several additional therapeutic classes have been explored as potential partners for CAR-T therapy in MM, largely supported by preclinical evidence but with limited or no clinical validation to date. Proteasome inhibitors such as bortezomib and carfilzomib have been shown in preclinical models to stabilize and upregulate BCMA expression on myeloma cells, thereby enhancing recognition and tumor clearance mediated by BCMA-directed CAR-T cells *in vivo* (192, 193). Despite this strong rationale, clinical validation of PI–CAR-T combinations in MM is currently lacking. A more advanced strategy to increase antigen density involves γ-secretase inhibition, as BCMA shedding by γ-secretase generates soluble BCMA that can limit the efficacy of BCMA-targeted therapies (179). Preclinical studies showed that γ-secretase inhibitors (GSIs) increase BCMA surface levels in myeloma cells, and enhance BCMA CAR-T recognition and cytotoxicity (194) and early clinical evaluation in the phase I study NCT03502577 showed that crenigacestat administered alongside BCMA CAR-T increased BCMA expression and induced deep responses with manageable toxicity (179). In addition, retrospective analyses suggested improved survival in BCMA-naïve patients with low baseline BCMA expression when GSIs were co-administered (195). Immune checkpoint pathways also represent a relevant target, as the immunosuppressive tumor microenvironment promotes CAR-T exhaustion through upregulation of PD-1/PD-L1, CTLA-4, TIM-3, and LAG-3 (112). While preclinical studies show that disrupting these inhibitory pathways enhances CAR-T proliferation, persistence, and antitumor activity (196, 197), clinical results with systemic PD-1 inhibitors combined with CAR-T have been inconsistent and appeared limited to selected patients with impaired T-cell fitness (198, 199). Consequently, the field is shifting toward intrinsic checkpoint modulation, as CAR-T cells engineered to resist PD-1 signaling demonstrate reduced exhaustion and superior tumor control in preclinical MM models (200). Notably, a first-in-human study of BCMA CAR-T cells incorporating PD-1 knockdown achieved an ORR of 85.7% with manageable toxicity in heavily pretreated RRMM (201). Prospective evaluation is ongoing in NCT04162119, assessing BCMA CAR-T cells secreting a PD-1–Fc fusion protein. Finally, epigenetic modulation with histone deacetylase inhibitors (HDACi) has emerged as another promising, yet still preclinical, approach. Preclinical studies show that HDAC inhibition enhances CAR-T fitness by promoting memory differentiation, strengthening effector functions, increasing TNF-α and IFN-γ production, and limiting exhaustion (202, 203). In MM, a functional CRISPR-based screening identified HDAC7 as a regulator of BCMA surface expression, and pharmacologic HDAC inhibition significantly increased BCMA density and CAR-T cytotoxicity (162). In parallel, HDACi also induce the pro-apoptotic protein NOXA, thereby sensitizing malignant plasma cells to CAR-T–mediated killing (163, 164). Although supportive data also exist from CD138-directed CAR-NK models, where entinostat improved CAR expression and *in vivo* tumor control (204), no clinical trials have yet reported efficacy outcomes for HDACi–CAR-T combinations in MM.

### 2.2 Combinational treatments based on immunotherapies in CLL

#### 2.2.1 Checkpoints inhibitors in combination therapies

Some clinical trials in CLL and RT have assessed the response to anti-PD-L1 (atezolizumab) and anti-PD-1 (nivolumab, pembrolizumab and tislelizumab) in combination with other drugs.

##### 2.2.1.1 Anti-PD-L1

Two main clinical studies have tested atezolizumab. The first one, the MOLTO trial, was a single-arm phase II trial to evaluate a combination of atezolizumab, venetoclax and obinutuzumab. This clinical trial included 28 patients who had a confirmed diagnosis of CLL or SLL with biopsy-proven transformation to diffuse large B-cell lymphoma variant of RT (DLBCL-RT) without previous treatment. The ORR of this study achieved 67.9% and CR was observed in the 28.6% of patients. PFS and OS at 12 months were 42.9% and 64.3% respectably. Regarding the immune system, the authors found during treatments significant decrease in CD3+, CD4+ and CD8+ T-cells counts as well as regulatory T cells (Treg). Moreover, in a *post-hoc* analysis they found that in patients in remission, the Th1/Th2 ratio was higher compared to the patients whose disease progressed (181). The second study was a multicentric phase I trial that enrolled 27 patients with relapsed or refractory DLBCL (rrDLBCL), including a transformed follicular lymphoma (FL) cohort and a RT cohort, which were treated with four cycles of R-GemOx (Rituximab, gemcitabine and oxaliplatin) and atezolizumab to evaluate the safety and efficacy of the treatment. Results were better in the FL cohort in comparison with the RT cohort, with an ORR of 79% and a CR of 43% in the transformed FL cohort and an ORR of 22% and a CR o 11% in the RT cohort (182). Overall, these studies suggest that atezolizumab-based combinations may have activity in RT patients and an immunoregulatory effect on T-cells, as seen in the first clinical trial, although results differ between the two studies due to their distinct clinical contexts (rrCLL and RT). Furthermore, the results do not support a clear advantage of chemotherapy-based combinations over targeted regimens in RT and further studies are needed to define the clinical utility of this combination regimen.

##### 2.2.1.2 Anti-PD-1

The use of nivolumab in combination therapies, mainly with the BTKi, ibrutinib, has been tested in clinical trials in both CLL and RT.

The first study of nivolumab and ibrutinib was a 1/2a phase including 144 BTKi-naïve patients, being 36 relapsed or refractory high-risk CLL or SLL and 20 RT. Although the efficacy of this combination was similar to ibrutinib as monotherapy in the CLL cohort with an ORR of 61%, a CR of 0% and a non-reached OS, of note, in RT patients, the results were more significant observing an ORR of 65%, a CR of 10% and an OS of 10.3 months (183). Thus, the combination of nivolumab with ibrutinib was suggested as an alternative therapy for RT patients and was further studied in a second clinical trial. This phase 2 study was carried out in 24 DLBCL-RT patients, which 13 of them had been already treated with BTKi. Among RT patients, only 10 out of 24 responded to the treatment. But 8 out of the 10 responders had a complete metabolic response (CMR) and the remaining 2 had a partial metabolic response (PMR) observed on PET scan. It was also observed that the response to this combination was much efficient in the BTKi-naïve subgroup (64% of patients responded) compared to the non-BTK-naïve group (23% of patients responded). Additionally, the median of OS was higher in patients who had not received any previous treatment for RT (24.1 months) compared to the already treated ones (9.1 months). Regarding CLL patients, it was less efficient, with only 2 out of 10 BTKi-naïve patients having a CR, but the study in this cohort was discontinued due to a slow accrual of participants (184). Overall, these data suggest that prior BTKi exposure may influence a response to the treatment, possibly reflecting both acquired resistance and the greater aggressiveness of RT. In CLL, the combination does not yet prove a synergistic effect, despite being described as potential in *in vivo* models (208) Recently, a different approach to the treatment was proposed based on the combination of nivolumab and copanlisib (PI3K inhibitor) in a phase I study. This clinical trial included 27 relapsed or refractory patients who had been already treated, which 14 of them had DLBCL-RT (1 excluded). Among RT patients, the ORR was of 31% (4 out of 13), with 2 patients with CR, but the PFS and OS were of 2.0 and 7.6 months, respectively. Despite these results, the 4 responding patients had a mean duration of treatment (DOR) of 15.2 months, and the CR ones remained in remission after a follow-up of 3.1 and 3.4 years. Furthermore, by integrating flow-cytometry and scRNA-seq data of blood samples from responding and non-responding RT patients, the authors reported a downregulation of PD1 expression, MYC and NF-kB signalling in T-cells after treatment in all patients and specifically, an enrichment of OXPhos and MYC targets routes pathways as well as a reprogramming towards Th1 phenotype in T-cells from responders (185).

Alternately, the effects of tislelizumab in combination with zanubrutinib, a second generation BTK inhibitor were studied in a phase 2 clinical study with 59 RT patients. The ORR was 58.3% including 18, 8% of CR, with a PFS of 10.0 months (186). Also, it was assessed in another phase 1/2 study with locally histologically confirmed RT, achieving an ORR of 61, 5% and 15, 4% of patients achieved CR (187).

In a phase I study, pembrolizumab was combined with dinaciclib, an inhibitor of CDK9, in patients with B-cell malignancies with one or more previous therapies given (17 rr CLL, 38 rrDLBCL and 17 RRMM). In rrCLL, the ORR was of 29.4% without CR patients and in rrDLBCL, the ORR was of 21.1% with 4 patients achieving CR (188). In another phase 2 study of the combination of pembrolizumab and ibrutinib in a cohort of 15 high risk rrCLL no advantage was observed in the combination compared to the use of monotherapy-ibrutinib except to the significant expansion of CD8+ T-cells in CLL patients (189).

Taken together, both combinations of nivolumab plus ibrutinib and tislelizumab plus zanubrutinib appear to be the most promising checkpoint inhibitor-based combinations in RT so far, particularly in BTKi-naïve patients, showing greater clinical activity than monotherapy BTKi or immune checkpoint inhibitors themselves. Noteworthy, nivolumab and ibrutinib combinational approach appears to be more efficient in patients without previous exposure to BTK inhibitors likely reflecting greater sensitivity to BTK inhibition than in previously treated patients. This observation opens the idea that alternative BTK-targeting strategies, including second generation BTK inhibitors such as zanubrutinib, or non-covalent BTK inhibitors such as pirtobrutinib could be explored in BTKi-acquired resistant patients. Moreover, combinations of BTKi with anti-PD1 agents have achieved more promising results that those with PI3K inhibitors (copanlisib), although the durable remissions observed in some complete responders treated with nivolumab plus copanlisib suggest that this approach may still benefit a selected subgroup. However, further research might be needed to better define the safety of these regimens and its immunoregulatory effects observed in RT patients. A deeper understanding of the immune landscape in RT will likely be essential for improving the efficacy of immunotherapies and for the rational design of future combination strategies.

#### 2.2.2 CAR-T cells in combination therapies

There are some clinical trials that describe combinational therapies involving CAR-T cells. For instance, the combination with ibrutinib was assessed in two different clinical trials. The first was a phase 2 clinical trial including 16 CLL patients treated with ibrutinib for six or more months who had not achieved CR, and who were subsequently dosed with anti-CD19 CAR-T cells (CART-19). This obtained a CR of 44%, and an estimated OS and PFS at 48 months of 84% and 70%, respectively. In addition, at 12 months, 72% of the patients had undetectable MRD (190). In another trial, a phase 1/2 study, 19 patients with rrCLL who had been inefficiently treated with ibrutinib (no CR achieved) enrolled CART-19 together with ibrutinib. Following iwCLL criteria, ORR at 4-week was 83% (CR = 22% and PR = 61%), with MRD-negative marrow responses by flow cytometry in 72% of patients OS and PFS at 12 months were of 64% and 38% respectably (191). These results suggest that ibrutinib may enhance CAR-T activity. Mechanistically, preclinical studies have shown that ibrutinib enhances CAR-T cells derived from CLL patients by increasing both their expansion and viability of these cells, while reducing T-cell exhaustion through inhibition of interleukin-2-inducible T-cell kinase (ITK) and downregulation of exhaustion markers such as PD-1, TIM-3 and LAG-3 (209). Additionally, in xenograft models, ibrutinib has been associated with enhanced T-cell activation and cytokine secretion (210) and may also reduce CAR-T related toxicity in CLL (211). However, from a clinical perspective, the available evidence remains limited by small cohort sizes, early-phase study design, restriction to rrCLL patients and the lack of current evaluation in RT patients.

Some preclinical studies have explored the use of PI3K inhibitors with CAR-T due to its role in T-cell-mediated autoimmunity. Based on this, some studies tried this combination and showed that PI3K inhibitors such as duvelisib or idelalisib (anti PI3Kδ/γ) could have metabolic reprogramming effects on CAR-T cells. One research showed that idelalisib increased mitochondrial activity and reduced exhaustion markers of CART-19 and in *in vivo* models, made a superior expansion of T-cells and increased killing of CLL cells (212). A second study also analysed the effects of duvelisib in CAR-T therapy in ex vivo models with CLL cells from patients and in *in vivo* with NOG mice. The main results were a normalisation of CD4+/CD8+ T-cell ratio, a decrease in TIM-3 and LAG-3 exhaustion markers in CD8+ T-cells, a potential epigenetic reprogramming of CART-19 towards a stem-like state and a potential increase in mitochondrial volume. Beside this, cytotoxicity, expansion and cell survival were far better in CAR-T cells accompanied with duvelisib in *in vivo* models (213). Altogether, these studies suggest that PI3K inhibition may play a role as a CAR-T adjuvant through immune reprogramming However, this strategy remains at the preclinical stage, and its clinical translation may be limited given the well-recognized toxicity associated with PI3K inhibitors.

#### 2.2.3 Bispecific antibodies in combination therapies

Bispecific antibodies are emerging as an alternative to the other mentioned immunotherapies. Some studies have shown results *in vitro* using patient-derived T-cells with bispecific antibodies in combination with other drugs. However, in contrast with MM, where BsABs have already been approved as a therapy, in CLL, they are still being evaluated in clinical trials and at the time this review is written, there are three on-going clinical trials recruiting patients to assess the effects of CD20xCD3 bispecific antibodies in combination with BTKi or PD-L1 inhibitors in RT patients or BCL2 and CD-20 inhibitors in CLL patients. The clinical trials are registered as NCT06043674 and NCT06735664 for RT ones and NCT07218510 for CLL ones.

*In vitro* studies showed that bispecific CD19xCD3 antibody in combination with ibrutinib promoted the autologous T-cells cytotoxicity against CLL cells, while reducing the CTLA-4 blockade, and differentiating the T-cells to a Th1 active phenotype in contrast to the T-cell treated without ibrutinib which displayed less cytotoxicity and activation due to its Th2 phenotype (214). The same authors also studied the *in vitro* effects of CD20xCD3 bispecific antibody epcoritamab in combination with venetoclax, using peripheral blood mononuclear cells from BTKi and non-BTKi treated patients to assess the cytotoxicity of the T-cells against CLL cells. The results showed that the combination was more effective than either agent alone. Epcoritamab promoted the expansion and activation of T-cells and the differentiation into Th1 cells and effector memory cells, whereas venetoclax induced superior killing of CLL cell (215). Additionally, the effects of CD20xCD3 bispecific antibody have been tested *in vivo* using Eµ-TCL1 mice. This bispecific has been shown that not only depleted the malignant cells in 80% of treated mice when it was combined with CAR-T cells, compared with 20% when CAR-T cells were used alone, but also enhanced expansion and activation of both endogenous CD8+ T-cells and anti-CD19 CAR-T cells, while increasing proliferation and cytotoxicity of endogenous T-cells (216).

Overall, these preclinical studies themselves show an important role of the bispecific antibodies (Both CD19xCD3 and CD20xCD3), in the activation and expansion of both endogenous T-cells and CAR-T cells. However, their effects have not yet been demonstrated in clinical trials, and their therapeutic value in patients remains to be established.

**Figure 2:**
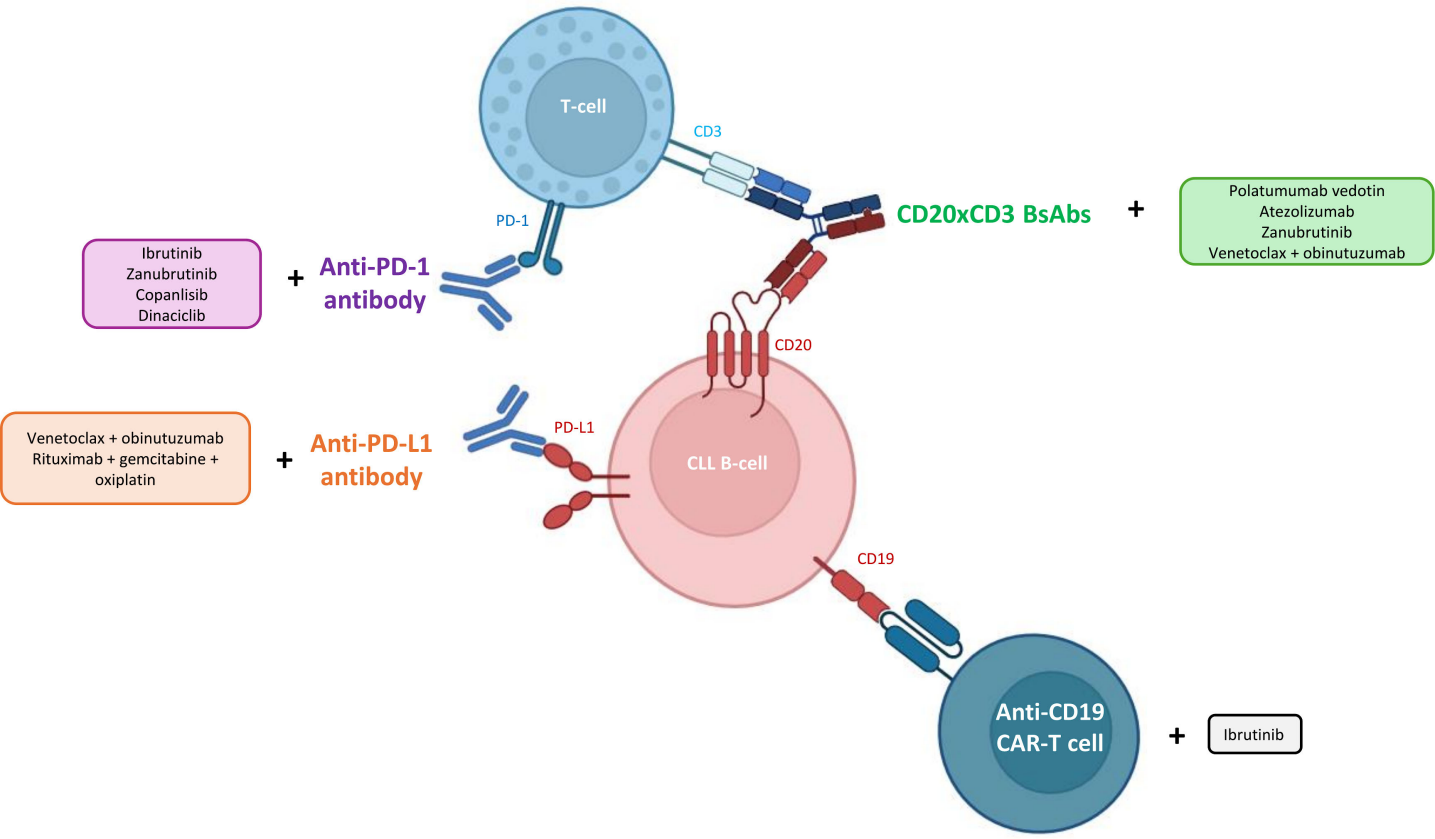
Immunotherapy in combinatorial therapies in CLL.

As shown in **Figure 2**, PD-1/PD-L1 inhibitors, CAR-T cells and BsAbs are shown in combination with the drugs described in the previous studies.

#### 2.2.4 Other potential combinations in preclinical models

Besides the advances obtained from clinical trials, some preclinical studies have postulated the beneficial effects of different combination therapies to be used in CLL.

In the context of the role of HDAC in immunobiology of cancers, CLL murine models reported the beneficial immunomodulatory effects of the inhibition of HDAC6 on CLL cells and its immunosuppression by the PD-1/PD-L1 blockade. Specifically, the authors genetic silenced and pharmacological inhibited HDAC6 in Eµ-TCL1 CLL murine model and they observed an immunoregulation in B-cells, with a reduction in the expression of PD-L1 on CLL B cells and IL-10 secreted levels, but an increase in the MHC-antigen presentation. Related to T-cells, they also observed a decrease in exhausted T and Treg cells and a change towards a Th1 phenotype in HDAC6-inhibited models. Based on these results, they evaluated *in vivo* the combination therapy of HDAC6 inhibition and PD-1/PD-L1 blockade improved the efficiency of anti PD-1/PD-L1 monoclonal antibodies in monotherapy. These results showed the rationale to assess HDAC6 inhibition together with immune checkpoint blockade for treatment in CLL (217). In another study, the authors explored the combination of HDAC inhibitors with CD19 CAR-T cells in B-cell models. They observed that pretreated cells with these inhibitors increased the cytotoxicity effects of CAR-T cells dependently on NOXA expression (218). These results were uncovered thanks to CRISPR screenings which represent a powerful tool to uncover novel therapeutic vulnerabilities to develop combinational therapies (219). In fact, another study using CRISPR screenings identified modulator genes of CD19 expression, highlighting NUDT21 as a repressor of CD19 expression. Therefore, its inhibition generated an increase of CD19 in cell surface which can increase sensitivity to CD19 CAR-T cells or BsAbs treatment (220).

As it has been showed, several genes might be potential therapeutic targets due its role in immunosuppression or regulation of antigen expression. Therefore, further clinical trials may be needed to be carried out in CLL and RT to explore these potential combinatorial immunotherapies. Of note, HDAC inhibitors had been shown as effective enhancers of immune checkpoint inhibitors and CAR-T cells in preclinical studies, so it could be interesting to carry out clinical trials to assess its effects in combination therapies.

## 3 Influence of the gut microbiota in immunotherapy

The human gut microbiota (GM) represents the body’s densest and most metabolically active ecosystem, hosting nearly 100 trillion microorganisms, predominantly bacteria (221). It participates in key physiological processes such as metabolic homeostasis and immune regulation. Dysbiosis, defined as loss of diversity, altered composition, or functional imbalance has emerged as a hallmark of cancer, driving chronic inflammation, immune evasion and tumor progression (222, 223). Beyond its contribution to tumorigenesis, the gut microbiota also modulates responses to multiple therapies, including chemotherapy, targeted therapy, and even immunotherapy (224).

Immunotherapies, have revolutionized cancer treatment, inducing durable clinical remissions and extended survival across several malignancies (225). However, their efficacy varies influenced by both tumor-intrinsic factors and host variables. Over the last decade, both clinical correlations and preclinical models have associated specific microbial signatures, including high alpha-diversity and distinct metabolomic profiles, with improved immunotherapy outcomes (226, 240).

Mechanistically, while much remains to be fully elucidated in humans, preclinical studies suggest that the microbiota shapes anti-tumor immunity through multiple interconnected pathways involving microbial metabolites, immune cell priming, and systemic inflammatory tone. Microbial bioactive products such as short-chain fatty acids (SCFAs, e.g., butyrate and propionate), secondary bile acids, and tryptophan catabolites directly influence immune cell states (227, 228). Specifically, SCFAs enhance CD8+ memory formation and promote dendritic cell co-stimulation via metabolic and epigenetic reprogramming, thereby reinforcing the immune programmes required for durable responses to ICIs and CAR-T therapies (229, 230). SCFAs display both antitumor and immunomodulatory effects. On the one hand, they can directly inhibit tumor proliferation and induce apoptosis (231). On the other hand, SCFAs enhance antitumor immunity by acting on multiple immune cell populations. They regulate dendritic cells and macrophages via HDAC inhibition (232, 233) and control the differentiation and suppressive function of FOXP3+ regulatory T-cells via both HDAC-dependent mechanisms and G-protein–coupled receptor signalling (234, 235). Importantly, SCFAs also promote cytotoxic CD8+ T-cell function by enhancing glycolysis and oxidative phosphorylation, increasing acetyl-CoA availability, and supporting IL-12-dependent effector responses, thereby improving antitumor activity (228, 230). However, their effects appear to be context-dependent, as elevated systemic levels of butyrate have been associated with reduced ICI efficacy, potentially due to excessive induction of regulatory T cells, highlighting the complexity of these interactions (236). Although the precise role of SCFAs in modulating immunotherapy responses remains incompletely defined, their pleiotropic effects on tumor biology, immune regulation, and cellular metabolism position them as key mediators of microbiota–immunotherapy interactions and promising targets for therapeutic intervention.

Other microbial metabolites, such as inosine, have also been linked to immunotherapy response. In particular, *Akkermansia muciniphila* and *Bifidobacterium pseudolongum* appear to enhance ICI efficacy through activation of the inosine-A2A receptor pathway, promoting T-cell activation and modulation of the tumor microenvironment via cAMP-PKA-CREB signalling (237).

In the context of ICIs, a robust correlation between gut composition and response is well-established across solid tumors where both preclinical and clinical studies have consistently shown that specific microbial signatures are associated with improved outcomes (237–239, 243). Mechanistically, this crosstalk involves enhanced antigen presentation, modulation of immune cell populations, metabolic reprogramming, and increased immune cell infiltration into the tumor microenvironment (TME). Early studies demonstrated that commensals like *Bifidobacterium* spp. have been shown to enhance anti-PD-1 efficacy by promoting dendritic cell maturation (238), while *Akkermansia muciniphila* and *Enterococcus hirae* have been associated with improved responses to PD-1/PD-L1 blockade through IL-12-dependent recruitment of T cells into the TME (240). Microbiota-driven metabolic pathways further contribute by modulating cytokine production (e.g., IFN-γ, IL-2) and T-cell function (241). Favorable microbial profiles have been associated with increased systemic immune activation, improved T-cell responses, higher frequencies of memory CD8+ T cells and NK cells (242–244). Functional evidence supports a causal role, as faecal microbiota transplantation (FMT) from responders increases DC frequency and augments T-cell responses (245). Clinically, responders exhibit higher microbial diversity and enrichment of SCFA-producing bacteria, including *Akkermansia muciniphila*, *Bifidobacterium longum*, and *Faecalibacterium prausnitzii*, while antibiotic exposure is consistently associated with inferior survival (240, 246).

Similarly, the efficacy of CTLA-4 blockade is influenced by the microbiota through immune and metabolic mechanisms. *Bacteroides* fragilis can restore responsiveness via IL-12-dependent Th1 priming (239). In clinical settings, enrichment in Firmicutes has been associated with improved progression-free and overall survival, potentially through increased ICOS expression on CD4+ T-cells and elevated CD25 levels, reflecting enhanced T-cell activation (247). However, microbiota-derived metabolites may exert context-dependent effects, as elevated systemic levels of butyrate have been associated with reduced efficacy of CTLA-4 blockade through inhibition of DC maturation and co-stimulatory signaling (236).

However, despite consistent associations across tumor types, results remain heterogeneous and no universal microbial signature has emerged as a reliable predictor of response; moreover, most mechanistic insights derive from solid tumor models, underscoring the need for further longitudinal and mechanistic studies, particularly in hematological malignancies.

Beyond ICIs, the gut microbiota also modulates the efficacy and toxicity of ACT, including CAR-T cells, through immune and metabolic mechanisms. Early studies showed that antibiotic-induced depletion of commensal bacteria impaired adoptive cell transfer (ACT) efficacy, partially recoverable by microbial ligands like LPS via TLR4 activation (248). Additionally, gut microbiota can promote the expansion and persistence of adoptively transferred cytotoxic T cells (CTLs) both in humans and mice (228).

Understanding these interactions is particularly urgent in hematological malignancies like MM and CLL where the convergence of immune dysfunction and dysbiosis likely underpins resistance to current immunotherapies.

### 3.1 Influence of the GM in immunotherapy response in MM

Accumulating evidence indicates that the GM plays an important role in MM pathogenesis via the “gut-bone marrow axis, ” influencing both systemic immunity and the tumor microenvironment (249). Dysbiosis in MM is characterised by depletion of SCFA-producing bacteria and an enrichment of nitrogen-recycling taxa. This creates a metabolic and immune environment that can modify disease progression and treatment response (231, 250). Metabolically, nitrogen-recycling bacteria such as *Klebsiella* and *Streptococcus* have the capacity to convert excess urea into L-glutamine, which can be reutilized by MM cells to fuel their metabolic demands (251). Immunologically, gut microbiota drives the expansion of IL-17-producing cells and eosinophils. These cells migrate to the bone marrow, thereby creating a pro-inflammatory microenvironment that supports the survival of malignant plasma cells and accelerates progression (252, 253). Furthermore, the loss of beneficial SCFA-producing *Faecalibacterium prausnitzii* and *Eubacterium hallii* results in reduced levels of immunoregulatory SCFAs, thereby promoting IL-6 and NF-κB signalling and reinforcing pro-tumor inflammatory environment (254, 255). These observations are further supported by recent data in myeloma precursor disorders showing that a high-fiber, plant-based dietary intervention can reshape the gut microbiota and increase butyrate-associated signatures. Even though the present study did not directly evaluate immunotherapy, it serves to reinforce the biological rationale for microbiota-directed strategies that are aimed at restoring SCFA production and enhancing the immune-metabolic context of MM (256). Collectively, these microbiota-driven alterations show how GM contributes to MM pathogenesis, but potentially can influence in therapeutic responses, including immunotherapies.

Microbiota-driven mechanisms offer a strategy to overcome the limited efficacy of ICIs in MM (257). In combination with PIs (**Figure 1**), there were cases of gastrointestinal toxicity, a phenomenon that may be linked to PI-induced gut injury and specific dysbiotic signatures. For instance, enrichment of *Bacteroides intestinalis*, has been associated with increased mucosal IL-1β and IL-17 in patients experiencing ICI-related gastrointestinal adverse event (257, 258). Dysbiosis characterised by a reduction in SCFA-producing bacteria, has been demonstrated to impair dendritic cell priming and promote T-cell exhaustion. This may provide a potential explanation for the observed lack of ICI efficacy (251). Interestingly, emerging data suggests that targeted modulation could reverse this predisposition. A recent study demonstrated that supplementation with *Prevotella melaninogenica* delayed the progression from SMM to active disease. This intervention increased SCFA production and restrained pathogenic Th17 polarization, thereby enhancing the efficacy of immune checkpoint blockade while mitigating toxicity (259). Although these findings are yet to be replicated in a clinical setting, they do support the concept that microbiota modulation may help uncouple efficacy from toxicity and potentially reopen the door for ICIs in MM.

Furthermore, in the context of hematological malignancies, including RRMM, longitudinal studies have demonstrated that the composition and diversity of the gut microbiota dynamically change during CAR-T therapy correlating with both efficacy and toxicity (205, 260), as shown in **Table 2**. Clinical evidence indicates that exposure to broad-spectrum antibiotics either before or around CAR-T infusion is associated with profound microbiome disruption, leading to impaired CAR-T expansion, reduced cytokine production, delayed hematologic recovery, and inferior clinical responses (261–264). These findings suggest that the preservation of microbial diversity and metabolic functionality is critical for sustaining CAR-T fitness and antitumor activity.

**Table 2:** Changes observed in gut microbiota in MM patients receiving CAR-T therapy.

Clinical association	Change	Taxa	Note	Ref.
Response to CAR-T	↑ Enriched	*Bifidobacterium(g), Prevotella(g), Sutterella(g), Oscillospira(g), Paraprevotella(g), and Collinsella(g)*,	Higher in CR vs PR (pre/post CAR-T)	(205)
	↑ Enriched	*Sutterella, Eggerthellaceae (f), Enterobacteriaceae (f)* *Eggerthellaceae, Enterobacteriaceae*	Prolonged PFS	(205, 206)
	↑ Enriched	*Bifidobacterium(g), Prevotella(g), Sutterella(g), Oscillospira(g), and Collinsella(g)*,	Higher in CR vs PR (pre/post CAR-T + post-chemotherapy)	(205)
	↑ Enriched	*Faecalibacterium(g), Roseburia(g), Ruminococcus(g)* *Barnesiellaceae (f)*	Enriched in CR vs PR post-infusion.	(205, 206)
	↑ Enriched	*Flavonifractor plautii, Bacteroides thetaiotaomicron, Bilophila wadsworthia, Alistipes onderdonkii, Parabacteroides, Dysosmobacter, and Enterocloster. Phascolarctobacterium faecium, Blautia faecis*	Baseline responders	(207)
	↑ Enriched	*F plautii, Dysosmobacter, and Phascolarctobacterium faecium*	Baseline responders; longer PFS	(207)
	↓ Depleted	*Clostridium scindens*	Baseline responders; longer PFS	(207)
	↑ Enriched	*B. thetaiotaomicron, Blautia faecis, Dysosmobacter*		(206)
No response to CAR-T	↑ Enriched	Lachnospiraceae (family): Lachnoclostridium (g), Faecalimonas(g), and Enterocloster(g) Streptococcaceae (family): Lactococcus (g), Streptococcus (g)	Observed at baseline	(207)
	↑ Enriched	*Monoglobaceae (f), Erysipelatoclostridiaceae (f)*		(207)
Longitudinal analysis	↑ Enriched	*Enterococcus (g), Lactobacillus(g), Actinomyces(g), Sutterella(g), Prevotella* *Firmicutes (phylum)* *Acidaminococcaceae, Akkermansiaceae, and Barnesiellaceae*	Time-course changes during CAR-T (late vs baseline)	(207)
	↓ Depleted	*Bifidobacterium, Lachnospira; Bacteroidetes(phylum)* *Erysipelatoclostridiaceae*	Time-course changes during CAR-T (late vs baseline)	(207)
Toxicity (CRS / severe toxicity	↑ Enriched	*Enterococcus (E. faecalis. E.lavalensis)* *Leuconostoc* *Lactococcus* *Erysipelatoclostridiaceae (f)* *Butyricicoccaceae (f)*	During high CRS	(205)
	↑ Enriched	*Bifidobacterium*	High before and during CRS	(205)
	↑ Enriched	*Butyricicoccus, Roseburia* spp. *Blautia* spp. *Ruminococcus bromii Anaerotruncus rubinfantis, Propionibacterium freudenreichii, Lacticaseibacillus rhamnosus Firmicutes: Bacili (class), Lactobacillales (order)*,	Low /No CRS	(205)

In MM specifically, r esponders to anti–BCMA CAR-T (ide-cel) exhibit enrichment of *Flavonifractor plautii, Bacteroides thetaiotaomicron*, *Blautia fecis*, *and Dysosmobacter* spp. (**Figure 1**). In contrast, non-responders show reduced diversity and greater microbiome disruption of metabolic networks (207). Similarly, integrated multi-omics analyses in patients treated with another BCMA-directed CAR-T (ARI0002h), identified a correlation between gut microbiota composition and function, and CAR-T cell phenotypes, persistence and clinical response. Notably, specific microbial metabolites, such as succinate, emerged as potential enhancers of CAR-T metabolic fitness and therapeutic efficacy (206). Safety profiles are also influenced by the gut microbiota. CAR-T infusion is associated with loss of beneficial commensals and expansion of facultative pathobionts such as *Enterococcus* and members of the *Proteobacteria* phylum (**Figure 1**)—which has been linked to increased rates of CRS and neurotoxicity (205).

Additional mechanistic support comes from other hematologic malignancies. Higher abundances of taxa such as *Ruminococcus*, *Bacteroides*, and *Faecalibacterium* have been associated with improved responses to CD19 CAR-T therapy (265). Furthermore, enrichment of SCFA-producing taxa, including members of the *Ruminococcaceae* and *Lachnospiraceae* families, has been shown to correlate with better efficacy. Conversely, expansion of facultative pathobionts such as *Enterococcus* or specific *Proteobacteria* has been associated with high-grade CRS and neurotoxicity (220–222). Moreover, preclinical studies further suggest that microbiota restoration strategies, such as *Akkermansia* supplementation, may improve CAR-T persistence and cytotoxicity by promoting dendritic-cell activation and memory T-cell differentiation (219).

Mechanistically, recent studies suggest that microbiota-derived metabolites may directly reprogram CAR-T cells. In patients with non-Hodgkin lymphoma treated with CD19 CAR-T cells, higher microbial diversity and butyrate-related signatures were associated with better progression-free survival, and ex vivo butyrate exposure induced phenotypic and transcriptomic changes linked to enhanced antitumor efficacy (266). In a similar manner, the microbial postbiotic pentanoate has been demonstrated to fuel CAR-T function through metabolic–epigenetic crosstalk, as its conversion to citrate and redistribution via ATP-citrate lyase supports histone acetylation and effector programs in CAR-T cells (267).

Consequently, while a proportion of the evidence derives from other hematological malignancies, it provides a plausible mechanistic framework by which microbial metabolites may enhance CAR-T persistence, fitness, and toxicity in MM. However, the available evidence remains largely preclinical or associative and still requires validation in prospective clinical studies.

Despite no studies have evaluated the GM in other immunotherapies such as bispecific antibodies or antibody–drug conjugates, the dependency of these therapies on T-cell activation and inflammatory signalling suggests that microbiota-mediated immune and metabolic pathways may indirectly influence their efficacy (227, 228).

Collectively, these findings position the gut microbiota as both a biomarker and a therapeutic target to optimize the efficacy, durability, and safety of MM immunotherapies.

### 3.2 Influence of the GM in immunotherapy in CLL

The number of studies exploring microbiota alterations in CLL is significantly lower than in other hematologic malignancies. However, given that CLL is an antigen-driven malignancy marked by profound immune dysfunction, chronic inflammation and T-cell exhaustion, the microbiota may influence both disease biology and therapeutic response. No clinical study has yet directly evaluated its impact on immunotherapy outcomes.

Early clinical observations reported reduced microbial diversity in CLL patients, indicating a consistent dysbiosis associated with immune dysfunction (268). Larger cohorts have identified an enrichment of *Firmicutes* and a depletion of *Bacteroidota*, a profile that correlates with adverse prognostic markers such as unmutated IGHV status and elevated CD38 expression (269). Integrative analysis combining patient samples with the Eµ-TCL1 mouse model identified decreased microbial diversity and enrichment of genera such as *Mucispirillum* sp. and *Parabacteroides* sp. in aggressive disease, supporting a pathogenic contribution of the gut microbiota in CLL progression (270). Notably, recent work in this model demonstrated that dysbiosis drives gut-barrier dysfunction and promotes systemic markers of T-cell exhaustion, whereas microbiota depletion or modulation can delay leukemic progression (271). These data suggest that the microbiota does not merely reflect the disease state but actively impacts in the disease.

Although no clinical studies have directly linked gut microbiota composition to immunotherapy outcomes CLL, accumulating evidence from other B-cell malignancies treated with anti-CD19 CAR-T cell therapy strongly supports this possibility. In DLBCL and acute lymphoblastic leukemia (ALL), high microbial diversity and an enrichment of SCFA-producing taxa (e.g., *Faecalibacterium prausnitzii* and *Bifidobacterium* spp.) correlate with enhanced CAR-T expansion, improved effector function and superior clinical responses. Conversely, dysbiosis and low microbial richness associate with reduced CAR-T persistence, inferior outcomes, and increased toxicity (205, 260, 266, 272). In CLL, where CAR-T cells often exhibit suboptimal persistence and high rates of exhaustion, the gut microbiota may represent a critical modifiable factor.

It is also worth mentioning that given the high antibiotic burden in CLL patients, this may partially explain the limited efficacy of ICIs and CAR-T in this population (261, 262).

A similar logic applies to bispecific T-cell engagers (BiTEs). These therapies rely entirely on the redirection of endogenous T cells to attack CD19+ or CD20+ leukemic cells. Given that the T-cell compartment in CLL is profoundly dysfunctional, microbiota-derived signals that govern T-cell metabolic fitness, such as butyrate-mediated epigenetic tuning could be unrecognized determinants of bispecific antibody efficacy (228). Addressing the “inflammatory tone” set by a dysbiotic gut may therefore be essential to maximize the cytotoxic potential of these antibodies and mitigate off-target toxicities (227, 273).

## 4 Future perspectives

Overall, the therapeutic landscape of MM has been transformed by immunotherapy, leading to unprecedented response rates and improved clinical outcomes, even in high-risk patients. However, in CLL the most relevant therapeutic advances have mainly resulted from targeted agents, particularly BTK inhibitors and the BCL-2 inhibitor venetoclax. In addition, anti-CD20 monoclonal antibodies, such as obinutuzumab and rituximab, remain part of standard therapeutic approaches, especially in fixed-duration venetoclax-based regimens. Venetoclax-obinutuzumab is commonly used in the first-line setting, whereas venetoclax-rituximab is used in relapsed/refractory disease. However, some immunotherapy-based combination strategies such as PD-1/PD-L1 inhibitors and CAR-T cells have shown encouraging results, especially in patients with RT. Nevertheless, drug resistance, limited durability of responses, toxicities, and interpatient heterogeneity continue to represent major limitations to long-term disease control.

Future efforts should prioritize the optimization of immunotherapeutic combinations based on a deeper understanding of tumor genetic background, immune dysfunction, and the tumor microenvironment, alongside the integration of robust predictive biomarkers to guide patient selection and treatment decisions. Treatment combinations aiming to overcome immune exhaustion, prevent antigen escape, and reshape the immunosuppressive microenvironment may enhance the depth and durability of responses while minimizing toxicity. In parallel, determining the optimal timing and sequencing of these therapies, rather than universal upfront combination, will be essential, particularly in elderly or frail patient populations.

Despite growing interest in the gut microbiota as a modulator of antitumor immunity and immunotherapy efficacy, significant challenges still limit its translation into clinical practice, particularly in B-cell malignancies such as multiple myeloma (MM) and chronic lymphocytic leukemia (CLL). Current knowledge remains fragmented and is based largely on preclinical models or correlative human studies, with most mechanistic insights extrapolated from solid tumours. In MM, where the clinical integration of advanced immunotherapies is rapidly expanding, the microbiota offers a more immediate translational opportunity. However, although the gut–bone marrow axis provides a compelling biological framework, it remains unclear which microbial taxa, metabolites, or immune pathways are the main determinants of response and toxicity, including immune-related adverse events such as cytokine release syndrome during CAR-T cell or bispecific antibody therapy. Conversely, the clinical projection for CLL is currently more limited, as direct evidence linking gut microbiota composition to immunotherapy outcomes is still scarce, making most mechanistic inferences indirect.

In general, most available studies have concentrated on the associations between microbial signatures and treatment outcomes, as opposed to establishing causality or identifying reproducible functional mechanisms. The identification of robust microbiota-based biomarkers is further complicated by several challenges, including interpatient variability, differences in sampling, antibiotic exposure, diet, disease stage, and treatment context. Nevertheless, accumulating evidence from selected cancer types suggests that microbiota-targeted strategies—including dietary interventions, probiotics, prebiotics, faecal microbiota transplantation, and microbiota-derived or engineered microbial products—may enhance treatment response and tolerability when combined with standard therapies. Consequently, future research should aspire to transcend the limitations of current descriptive microbiome profiling methodologies, instead focusing on the implementation of longitudinal, functional, and prospective studies. These studies should integrate immune profiling, microbiome characterisation, and multi-omic approaches, with the objective of enabling patient stratification, treatment selection, and response monitoring. In this context, innovative clinical trial designs incorporating immunological and microbiome-related endpoints will be essential to develop personalized microbiota-modulating strategies and ultimately optimize immunotherapy outcomes in MM and, in the longer term, CLL, particularly in patients with refractory or treatment-resistant disease.
